# Intrathoracic appendicitis within a left-sided Bochdalek hernia: a case report

**DOI:** 10.1093/jscr/rjaf865

**Published:** 2025-10-31

**Authors:** Jasser Rchidi, Yassine Kallel, Ghazi Laamiri, Houda Gazzah, Mahdi Bouassida, Hassen Touinsi, Siwar Sbaihi, Mahmoud Derbel, Fatma Gabsi, Khaled Bouzaidi

**Affiliations:** Department of General Surgery, Mohamed Taher Maamouri University Hospital, Ezzedine Chelbi Avenue, Hammamet Nabeul 8000, Tunisia; Department of General Surgery, Mohamed Taher Maamouri University Hospital, Ezzedine Chelbi Avenue, Hammamet Nabeul 8000, Tunisia; Department of General Surgery, Mohamed Taher Maamouri University Hospital, Ezzedine Chelbi Avenue, Hammamet Nabeul 8000, Tunisia; Department of General Surgery, Mohamed Taher Maamouri University Hospital, Ezzedine Chelbi Avenue, Hammamet Nabeul 8000, Tunisia; Department of General Surgery, Mohamed Taher Maamouri University Hospital, Ezzedine Chelbi Avenue, Hammamet Nabeul 8000, Tunisia; Department of General Surgery, Mohamed Taher Maamouri University Hospital, Ezzedine Chelbi Avenue, Hammamet Nabeul 8000, Tunisia; Department of Radiology, Mohamed Taher Maamouri University Hospital, Ezzedine Chelbi Avenue, Hammamet Nabeul 8000, Tunisia; Department of Radiology, Mohamed Taher Maamouri University Hospital, Ezzedine Chelbi Avenue, Hammamet Nabeul 8000, Tunisia; Department of Radiology, Mohamed Taher Maamouri University Hospital, Ezzedine Chelbi Avenue, Hammamet Nabeul 8000, Tunisia; Department of Radiology, Mohamed Taher Maamouri University Hospital, Ezzedine Chelbi Avenue, Hammamet Nabeul 8000, Tunisia

**Keywords:** Bochdalek hernia, appendicitis, diaphragmatic hernia, incarcerated bowel

## Abstract

Bochdalek hernias are rare congenital defects usually diagnosed in infancy, and their presentation in adults is uncommon. Even rarer is the occurrence of acute appendicitis within a Bochdalek hernia; to date, only two adult cases have been reported, both involving right-sided or unspecified hernias. We describe a 21-year-old previously healthy man who presented with sudden left-sided chest pain and mild respiratory symptoms. Imaging identified a left posterolateral diaphragmatic hernia containing bowel loops and an inflamed appendix. He underwent emergency laparotomy with appendectomy and primary repair of the diaphragmatic defect without mesh due to contamination risk. Histopathology confirmed acute suppurative appendicitis. The patient’s recovery was uneventful, and he remained well at follow-up. This case underscores the diagnostic challenge posed by atypical thoracic presentations of abdominal pathology and highlights the critical role of computed tomography in enabling timely surgical intervention.

## Introduction

Congenital diaphragmatic hernia is a condition that occurs in ~1 in 2500 newborns, with a survival rate of 67% [1]. Bochdalek hernia is a diaphragmatic hernia that occurs due to a congenital defect in the posterolateral attachment of the diaphragm. It results from incomplete closure of the pleuroperitoneal membrane during fetal development. Although usually located on the left side, it may also occur on the right. During the perinatal period, Bochdalek hernia causes severe respiratory complications. In adults, it is extremely rare, with an estimated incidence of only 0.17% [2].

Adult Bochdalek hernias are frequently asymptomatic but may present with gastrointestinal or respiratory symptoms depending on the degree of organ herniation and thoracic compromise [3, 4]. To date, only two published cases in adults have reported acute appendicitis within a Bochdalek hernia [5, 6]. Both involved right-sided or unspecified hernias, and in one case, the patient had marfanoid features. To the best of our knowledge, this is the first documented case of left-sided intrathoracic appendicitis in an otherwise healthy adult male.

We present an uncommon thoracoabdominal emergency involving acute appendicitis within a Bochdalek hernia, associated with intrathoracic incarcerated bowel.

## Case report

A 21-year-old male presented to the emergency department with sudden onset of left-sided chest pain for the past two days. He had no surgical or trauma history, maintained regular bowel habits, and denied abdominal complaints, such as vomiting, nausea, diarrhea, or constipation. Vital signs were stable: blood pressure 135/72 mmHg, heart rate 64 bpm, and temperature 38.4°C. He had mild tachypnea, with an oxygen saturation of 97%. Clinical examination revealed reduced breath sounds in the left hemithorax. The abdomen was soft with mild tenderness and no signs of peritonitis.

Laboratory investigations showed leukocytosis (white blood cell count 17 000/μl) with neutrophilia and a markedly elevated C-reactive protein (240 mg/L). Abdominal ultrasound showed splenomegaly but no evidence of biliary pathology or intra-abdominal abscess.

**Figure 1:**
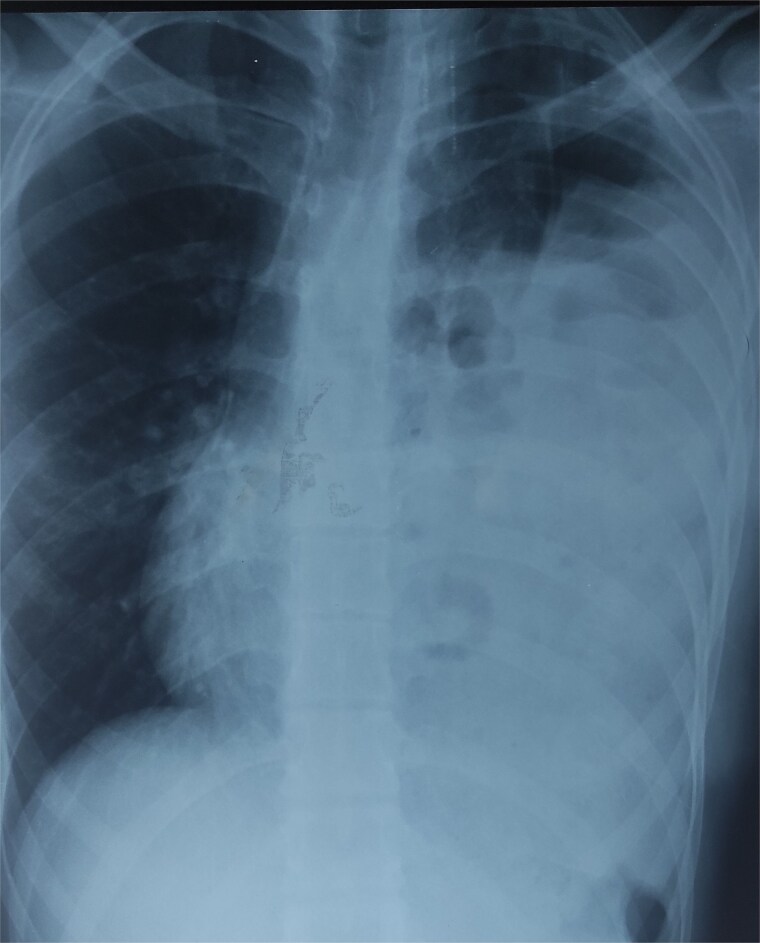
Chest X-ray showing air-filled bowel loops within the left hemithorax.

**Figure 2:**
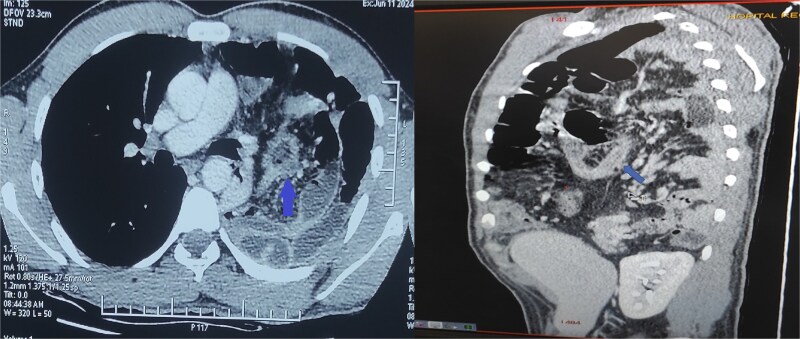
Contrast-enhanced CT of the chest and abdomen showing a left posterolateral diaphragmatic hernia with herniation of bowel and omentum into the thorax. The thickened appendix with surrounding fat stranding indicates acute appendicitis.

The differential diagnoses initially included pneumonia, pleural effusion, pericarditis, and hydropneumothorax. Electrocardiogram was normal. Chest X-ray revealed air-filled bowel loops in the left hemithorax (Fig. 1). A contrast-enhanced computed tomography (CT) scan of the chest and abdomen confirmed a left-sided posterolateral diaphragmatic defect with herniation of omentum, small bowel loops, appendix, caecum, ascending colon, and transverse colon into the thoracic cavity. The appendix appeared thickened with surrounding fat stranding, suggesting acute appendicitis. There was also left-sided reactive pleural effusion (Fig. 2).

**Figure 3:**
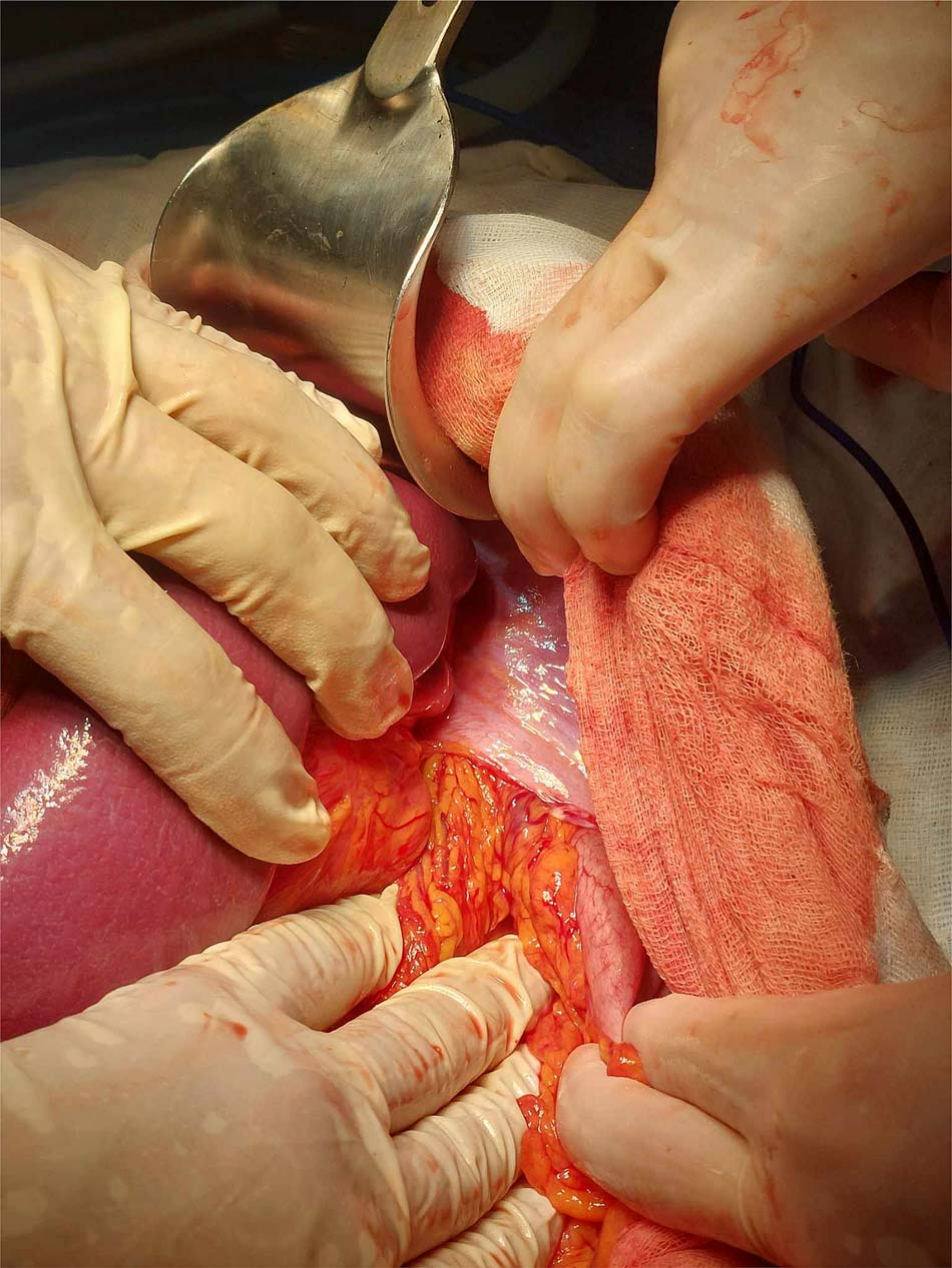
Left posterolateral diaphragmatic defect with herniated bowel and omentum.

A multidisciplinary decision was made to proceed with emergency surgery. The patient underwent an L-shaped midline-to-subcostal laparotomy under general anesthesia. Intraoperatively, a 6 cm left posterolateral diaphragmatic defect was visualized (Fig. 3). The hernia sac contained ~50 cm of incarcerated terminal ileum and inflamed appendix, covered with fibrinous exudate. There was no necrosis or perforation of the bowel. Due to dense adhesions and the large volume of displaced viscera, open surgery was favored over laparoscopy to ensure safe reduction and full inspection of the bowel.

**Figure 4:**
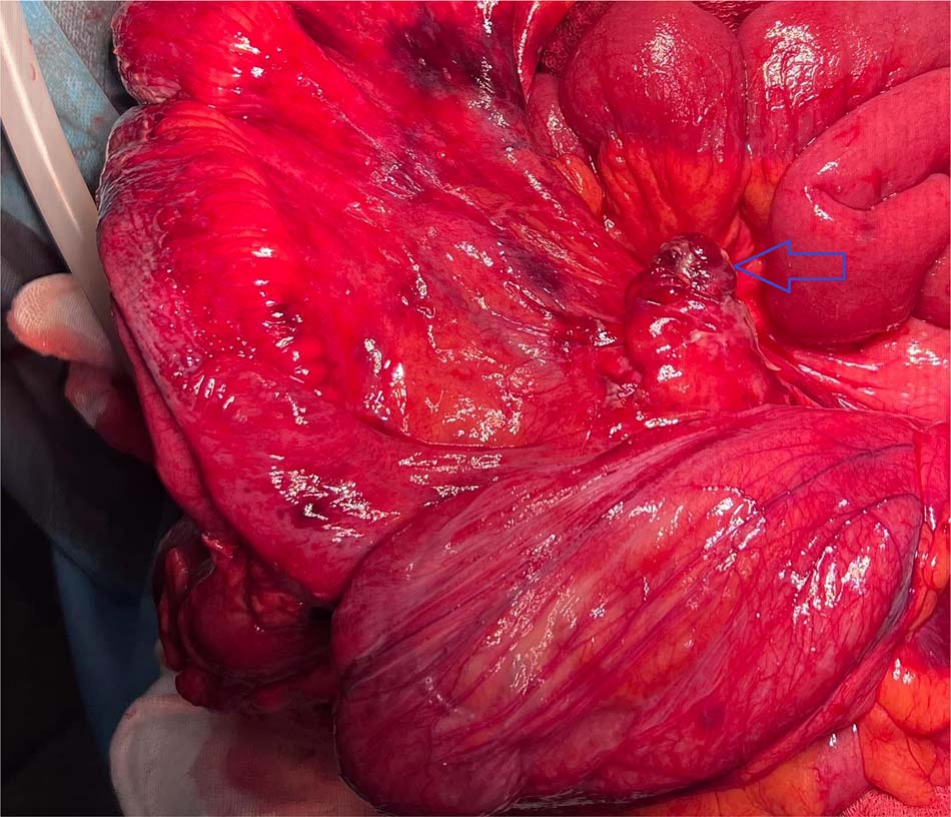
Hernia sac containing small bowl with the inflamed appendix.

The appendix was grossly inflamed, edematous, and necrotic, measuring ~10 mm in diameter (Fig. 4). An appendectomy was performed. The diaphragmatic defect was repaired with non-absorbable sutures. A synthetic mesh was not used due to the contaminated field and the risk of postoperative infection. A 28 Charrière chest tube was inserted into the left thoracic cavity. The patient was extubated the following day and started on broad-spectrum antibiotics for seven days.

Histopathological analysis of the resected appendix confirmed acute suppurative appendicitis without evidence of perforation or neoplasia.

The patient had an uneventful postoperative course. The chest tube was removed on day 5, and he was discharged in stable condition on day 8. At three-month follow-up, the patient remained asymptomatic with no clinical or radiological signs of recurrence. Follow-up imaging was not performed, as the patient declined further radiologic studies and was monitored with physical examination and inflammatory markers.

## Discussion

This case illustrates an extremely rare presentation of acute appendicitis within a Bochdalek hernia in an adult patient. Bochdalek hernias result from a failure of fusion between the septum transversum and pleuroperitoneal folds during fetal development. They are predominantly left-sided (85%) and usually diagnosed in infancy. In adults, the condition is rare and often discovered incidentally or during evaluation for nonspecific symptoms [2, 7, 8].

To date, only two adult cases of intrathoracic appendicitis have been reported in the literature [5, 6]. One involved a marfanoid patient with gangrenous appendicitis; the other was a right-sided diaphragmatic hernia. Our patient represents the first documented case of a left-sided Bochdalek hernia containing an inflamed appendix and small bowel loops in an otherwise healthy adult male.

Intrathoracic appendicitis can present as chest pain or respiratory symptoms, often mimicking pneumonia or pleural effusion. Studies have shown that 25%–38% of patients with adult congenital diaphragmatic hernias are initially misdiagnosed, sometimes resulting in inappropriate chest tube placement [9–12]. In our case, early CT imaging allowed for accurate diagnosis and avoided such complications.

CT remains the gold standard for diagnosing diaphragmatic hernias. It provides critical information on the location and size of the defect and the nature of herniated contents [13]. Recognition of atypical presentations is essential to avoid delays in diagnosis and management.

There is no consensus on the ideal surgical approach for congenital diaphragmatic hernias in adults. Laparoscopic repair is increasingly adopted in elective cases, while laparotomy remains preferred in emergencies or when bowel ischemia is suspected [14]. In our case, laparotomy was chosen due to the risk of incarceration, inflammation, and the need for complete adhesiolysis.

Mesh repair is avoided in infected or contaminated fields due to the risk of erosion and fistula formation. Although mesh use may reduce recurrence rates, non-mesh primary suture repair remains acceptable in emergent settings when the field is inflamed or contaminated [15].

## Conclusion

This case highlights a rare thoracoabdominal emergency: acute appendicitis within a left-sided Bochdalek hernia in an adult. The absence of classic abdominal symptoms and the presence of atypical chest pain contributed to diagnostic complexity. CT imaging was essential for accurate diagnosis. Open surgery enabled safe reduction of herniated viscera, appendectomy, and diaphragmatic repair without complications. Histopathology confirmed the diagnosis. Clinicians should maintain a high index of suspicion for congenital diaphragmatic hernia in young adults with unexplained chest symptoms. Prompt recognition and surgical intervention are key to avoiding morbidity.

## Patient perspective

The patient expressed satisfaction with the surgical outcome and experienced a good recovery.
